# Supplementary material for: Quinolone Resistance of Actinobacillus pleuropneumoniae Revealed through Genome and Transcriptome Analyses
Source: Int J Mol Sci. 2021 Sep 17;22(18):10036. doi: 10.3390/ijms221810036 (PMC8472844; doi:10.3390/ijms221810036)
Supplement: Supplementary file 1 [file ijms-22-10036-s001.zip › ijms-1353640-supplementary/ijms-1353640-SM final/ijms-1353640-SM 2/Supplementary Figure/supplementary Figure.pdf]

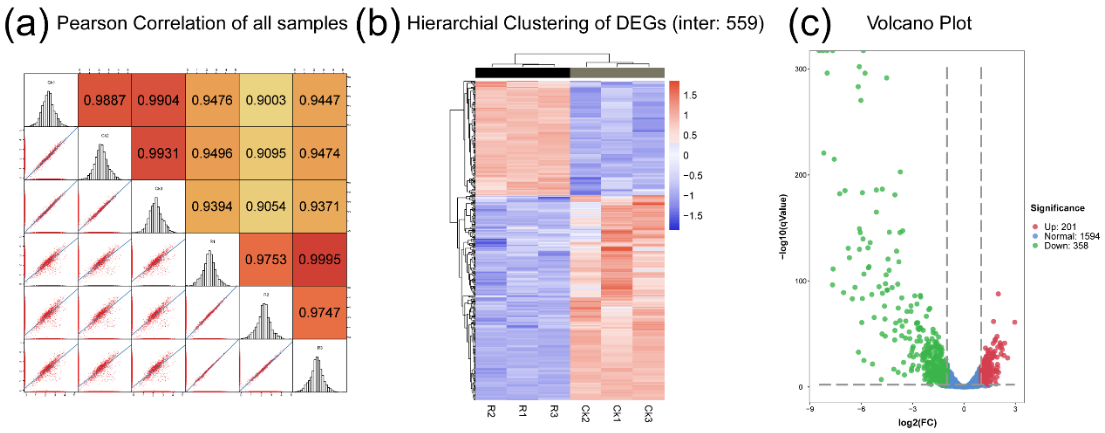

Supplementary Figure S1

RNA sequencing analysis. (a) Pearson correlation of all sample. (b) Heatmap showing gene expression patterns. (c) Volcano plots shows the difference of expression level between the wild and mutant strains.
