# Supplementary material for: Quinolone Resistance of Actinobacillus pleuropneumoniae Revealed through Genome and Transcriptome Analyses
Source: Int J Mol Sci. 2021 Sep 17;22(18):10036. doi: 10.3390/ijms221810036 (PMC8472844; doi:10.3390/ijms221810036)
Supplement: Supplementary file 1 [file ijms-22-10036-s001.zip › ijms-1353640-supplementary/ijms-1353640-SM final/ijms-1353640-SM 2/Supplementary Table/supplementary Table.pdf]

Table S1 Oligonucleotide primers used in this study.

Primers	Sequence（5’-3’）	Target gene	Product size(bp)	Original
gryA1-F	ATGCGATGTCTGTGATTGTC	gyrA	878	This study
gyrA1-R	TAATACCACTTCACCCACTG	71-946		
gryA2-F	GCGTGAAGGTTTATTAGCTC	gyrA	1044	This study
gyrA2-R	TCCGCTTCCTCGTTTTCAAT	1215-2239		
gryB-F	GTGTAGTGAAGAAAGCGGAT	gyrB	1012	This study
gyrB-R	TTCGCCACAAACTGCTCATT	917-1928		
parC1-F	GCTTCGCCTTCAAAAATCAT	parC	1080	This study
parC1-R	CGCTTTACCCGCCTTATTGC	796-1873		
parC2-F	TAACCCCAACCGAAGATGTG	parC	699	This study
parC2-R	ATCCTTTTCTTGCTCGTTCG	1496-2175		
parC3-F	TGCCGTTTATCGGTGACG	parC	567	[1]
parC3-R	CTGCCTCAGTCGGGTAAT	71-637		
parE-F	GGAAGGTGGCGAGCTATTGG	ParE	555	This study
parE-R	CCTCGTAACGGCAAAATCGC	774-1328		
parE2-F	TCTCATCGGCGCAAAGTCGT	parE	408	[1]
parE2-R	GGCGCAAGAACAAGGCACAT	1121-1528		

Table S2 Primers used for qRT-PCR

Primers	Sequence（5’-3’）	Target gene	Product sizep (bp)	Original
LamB_F	TTGGAAAGATGGCTCGTTAG	DRF63_RS06720	209	This study
LamB_R	CTTGTGCGTGACCATTATTC	maltoporin		
OmpP2A-F	GGTTCCGCTCGTGTAAAATG	DRF63_RS00030	139	This study
OmpP2A-R	TGCCGAGTTGTTGAGTTAAG	ompP2A		
OmpP2B-F	GTAATGCGAGAGGTGAAGTT	DRF63_RS03480	121	This study
OmpP2B-R	TTAGAACGACCGATACCACC	ompP2B		
OmpW-F	GCGATGTGATTTTCCGTGCC	DRF63_RS05955	1231	This study
OmpW-R	TTACTTTTGCCACTTCGCCT	ompW		
ApfA-F	CGGATGTCGAGATCTGCATATATAA	DRF63_RS04675	92	[2]
ApfA-R	CTGTCTAAGCTCCGTCATTTTTCTG	apfA		
ApfC-F	TTGGCGAAATCGGAAATACAG	DRF63_RS04665	86	[2]
ApfC-R	CGATTTACCGCAATATTTTGCA	apfC		
ComEA-F	CGAACAAAACTGCCGATAAACA	DRF63_RS08060	80	[2]
ComEA-R	TCCCACCAGTTTGGATTTAATTTC	comEA		
TonB1-F	TGGAAGCAAATCAACCTATGGA	DRF63_RS08640	150	[2]
TonB1-R	TCGCATTGCATTGCCATAA	tonB1		
ExbD1-F	TGAGAAAAAAGATGAGCCAAAAGA	DRF63_RS08630	83	[2]
ExbD1-R	CATTCACTAGCTTCTCGCCTAAATAA	exbD1		

rbsB-F	GAAAAAAGCCAAAGACCTCG	DRF63_RS09165	128	This study
rbsB-R	CTGTCGGGTTGATGAGTAAT	rbsB		
rbsC-F	CCTTTCGGTCGGTTCGGTCT	DRF63_RS09160	199	This study
rbsC-F	GCCACGCAATAACAACATCG	rbsC		
MATE-F	GGTTTACCGATCGGCTTTGC	DRF63_RS01980	100	This study
MATE-R	GATGACTTGCCACCACTTGC	MATE		
TolC-F	ATGAACAGACGGTGACCAGC	DRF63_RS01355	95	This study
TolC-R	TGCTTGTTGTAGGGTCGCAT	TolC		
MFS-F	ATCCTCACTCTTGCCGTTTT	DRF63_RS10025	105	This study
MFS-R	GCGGGTTTTGTTCGGGTAAT	MFS		
AcrB-F	ACGATTTCCATTGTCACGGT	DRF63_RS03195	96	This study
AcrB-R	CGCTACCGCTTCTTCAATTT	AcrB/AcrD/AcrF		
16s	GGAATAACTGGGCGTAAAGG		200	[3]
16s	GCTCAGTACATTCCCAAGG	16s		

[1] Wang YC, Chan JP, Yeh KS, et al. Molecular characterization of enrofloxacin resistant Actinobacillus pleuropneumoniae isolates[J]. Vet Microbiol, 2010, 142(3-4):309-312.

[2] Li L, Xu Z, Zhou Y, et al. Analysis on Actinobacillus pleuropneumoniae LuxS regulated genes reveals pleiotropic roles of LuxS/AI-2 on biofilm formation, adhesion ability and iron metabolism[J]. Microb Pathog, 2011, 50(6):293-302.

[3] Hathroubi S, Hancock MA, Bossé JT, et al. Surface Polysaccharide Mutants Reveal that Absence of O Antigen Reduces Biofilm Formation of Actinobacillus pleuropneumoniae[J]. Infection and immunity, 2016, 84(1).
