# Supplementary material for: Quinolone Resistance of Actinobacillus pleuropneumoniae Revealed through Genome and Transcriptome Analyses
Source: Int J Mol Sci. 2021 Sep 17;22(18):10036. doi: 10.3390/ijms221810036 (PMC8472844; doi:10.3390/ijms221810036)
Supplement: Supplementary file 1 [file ijms-22-10036-s001.zip › ijms-1353640-supplementary/ijms-1353640-SM/ijms-1353640-SM.pdf]

Table S1. Oligonucleotide primers used in this study.

Primers	Sequence (5′-3′)	Target Gene	Product Size(bp)	Original
gryA1-F<br>gyrA1-R	ATGCGATGTCTGTGATTGTC<br>TAATACCACTTCACCCACTG	*gyrA*<br>*71-946*	878	This study
gryA2-F<br>gyrA2-R	GCGTGAAGGTTTATTAGCTC<br>TCCGCTTCCTCGTTTTCAAT	*gyrA*<br>*1215-2239*	1044	This study
gryB-F<br>gyrB-R	GTGTAGTGAAGAAAGCGGAT<br>TTCGCCACAAACTGCTCATT	*gyrB*<br>*917-1928*	1012	This study
parC1-F<br>parC1-R	GCTTCGCCTTCAAAAATCAT<br>CGCTTTACCCGCCTTATTGC	*parC*<br>*796-1873*	1080	This study
parC2-F<br>parC2-R	TAACCCCAACCGAAGATGTG<br>ATCCTTTTCTTGCTCGTTCG	*parC*<br>*1496-2175*	699	This study
parC3-F<br>parC3-R	TGCCGTTTATCGGTGACG<br>CTGCCTCAGTCGGGTAAT	*parC*<br>*71-637*	567	[18]
parE-F<br>parE-R	GGAAGGTGGCGAGCTATTGG<br>CCTCGTAACGGCAAAATCGC	*ParE*<br>*774-1328*	555	This study
parE2-F<br>parE2-R	TCTCATCGGCGCAAAGTCGT<br>GGCGCAAGAACAAGGCACAT	*parE*<br>*1121-1528*	408	[18]

Table S2. Primers used for qRT-PCR.

Primers	Sequence (5′-3′)	Target Gene	Product Sizep (bp)	Original
LamB_F<br>LamB_R	TTGGAAAGATGGCTCGTTAG<br>CTTGTGCGTGACCATTATTC	*DRF63_RS06720*<br>*maltoporin*	209	This study
OmpP2A-F<br>OmpP2A-R	GGTTCCGCTCGTGTAAAATG<br>TGCCGAGTTGTTGAGTTAAG	*DRF63_RS00030*<br>*ompP2A*	139	This study
OmpP2B-F<br>OmpP2B-R	GTAATGCGAGAGGTGAAGTT<br>TTAGAACGACCGATACCACC	*DRF63_RS03480*<br>*ompP2B*	121	This study
OmpW-F<br>OmpW-R	GCGATGTGATTTTCCGTGCC<br>TTACTTTTGCCACTTCGCCT	*DRF63_RS05955*<br>*ompW*	1231	This study
ApfA-F<br>ApfA-R	CGGATGTCGAGATCTGCATATATAA<br>CTGTCTAAGCTCCGTCATTTTTCTG	*DRF63_RS04675*<br>*apfA*	92	[23]
ApfC-F<br>ApfC-R	TTGGCGAAATCGGAAATACAG<br>CGATTTACCGCAATATTTTGCA	*DRF63_RS04665*<br>*apfC*	86	[23]
ComEA-F<br>ComEA-R	CGAACAAAACTGCCGATAAACA<br>TCCCACCAGTTTGGATTTAATTTC	*DRF63_RS08060*<br>*comEA*	80	[23]
TonB1-F<br>TonB1-R	TGGAAGCAAATCAACCTATGGA<br>TCGCATTGCATTGCCATAA	*DRF63_RS08640*<br>*tonB1*	150	[23]
ExbD1-F<br>ExbD1-R	TGAGAAAAAAGATGAGCCAAAAGA<br>CATTCACTAGCTTCTCGCCTAAATAA	*DRF63_RS08630*<br>*exbD1*	83	[23]
rbsB-F<br>rbsB-R	GAAAAAAGCCAAAGACCTCG<br>CTGTCGGGTTGATGAGTAAT	*DRF63_RS09165*<br>*rbsB*	128	This study
rbsC-F<br>rbsC-F	CCTTTCGGTCGGTTCGGTCT<br>GCCACGCAATAACAACATCG	*DRF63_RS09160*<br>*rbsC*	199	This study
MATE-F<br>MATE-R	GGTTTACCGATCGGCTTTGC<br>GATGACTTGCCACCACTTGC	*DRF63_RS01980*<br>*MATE*	100	This study
TolC-F<br>TolC-R	ATGAACAGACGGTGACCAGC<br>TGCTTGTTGTAGGGTCGCAT	*DRF63_RS01355*<br>*TolC*	95	This study

MFS-F	ATCCTCACTCTTGCCGTTTT	*DRF63_RS10025*	105	This study
MFS-R	GCGGGTTTTGTTCGGGTAAT	*MFS*		
AcrB-F	ACGATTTCCATTGTCACGGT	*DRF63_RS03195*	96	This study
AcrB-R	CGCTACCGCTTCTTCAATTT	*AcrB/AcrD/AcrF*		
16s	GGAATAACTGGGCGTAAAGG		200	[46]
16s	GCTCAGTACATTCCCAAGG	*16s*		

Table S3. Genomic drug-resistant efflux pump.
