# Supplementary figures and images for: Quinolone Resistance of Actinobacillus pleuropneumoniae Revealed through Genome and Transcriptome Analyses
Source: Int J Mol Sci. 2021 Sep 17;22(18):10036. doi: 10.3390/ijms221810036 (PMC8472844; doi:10.3390/ijms221810036)

**(a)** Pearson Correlation of all samples

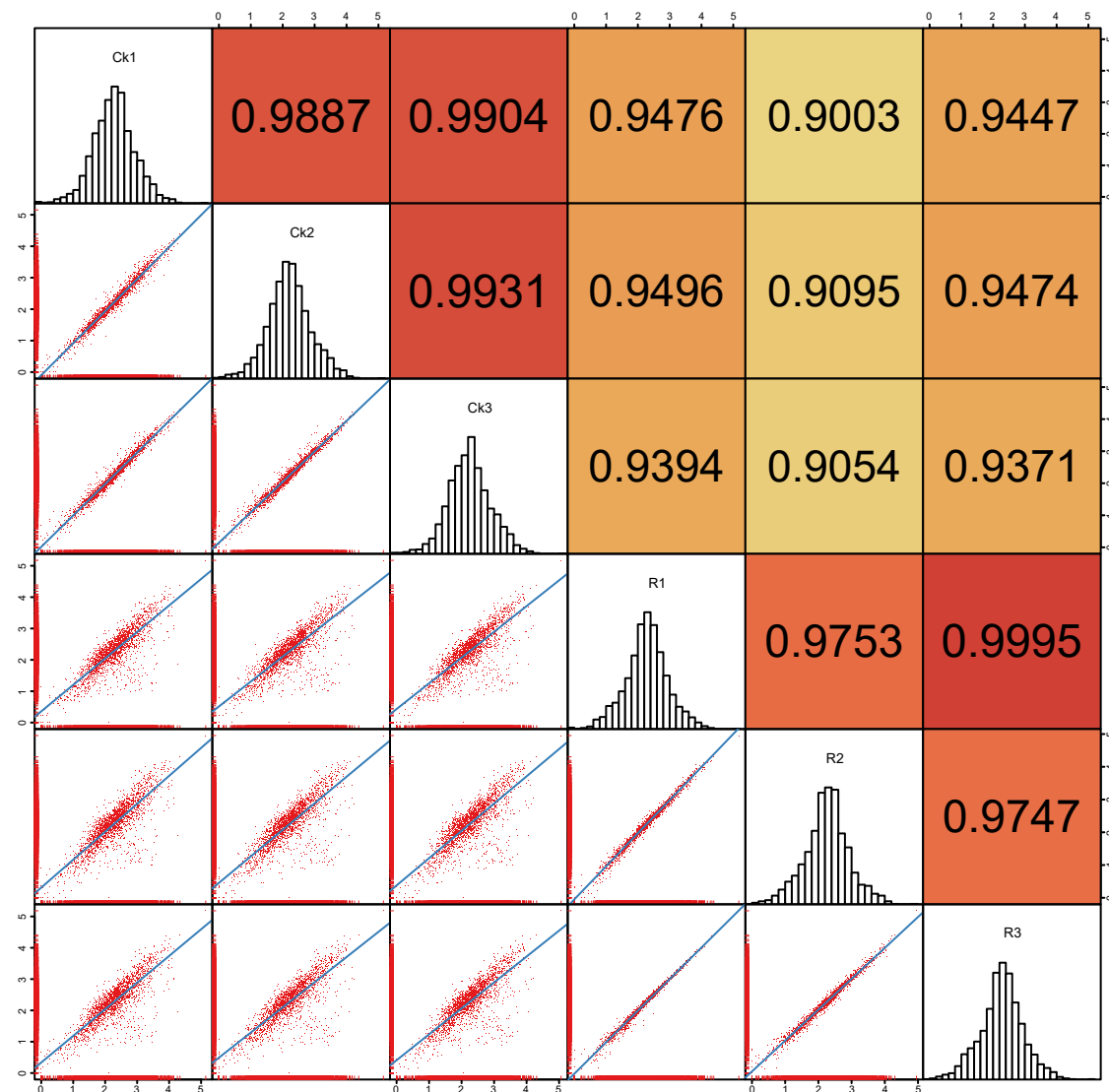

**(b)** Hierarchical Clustering of DEGs (inter: 559)

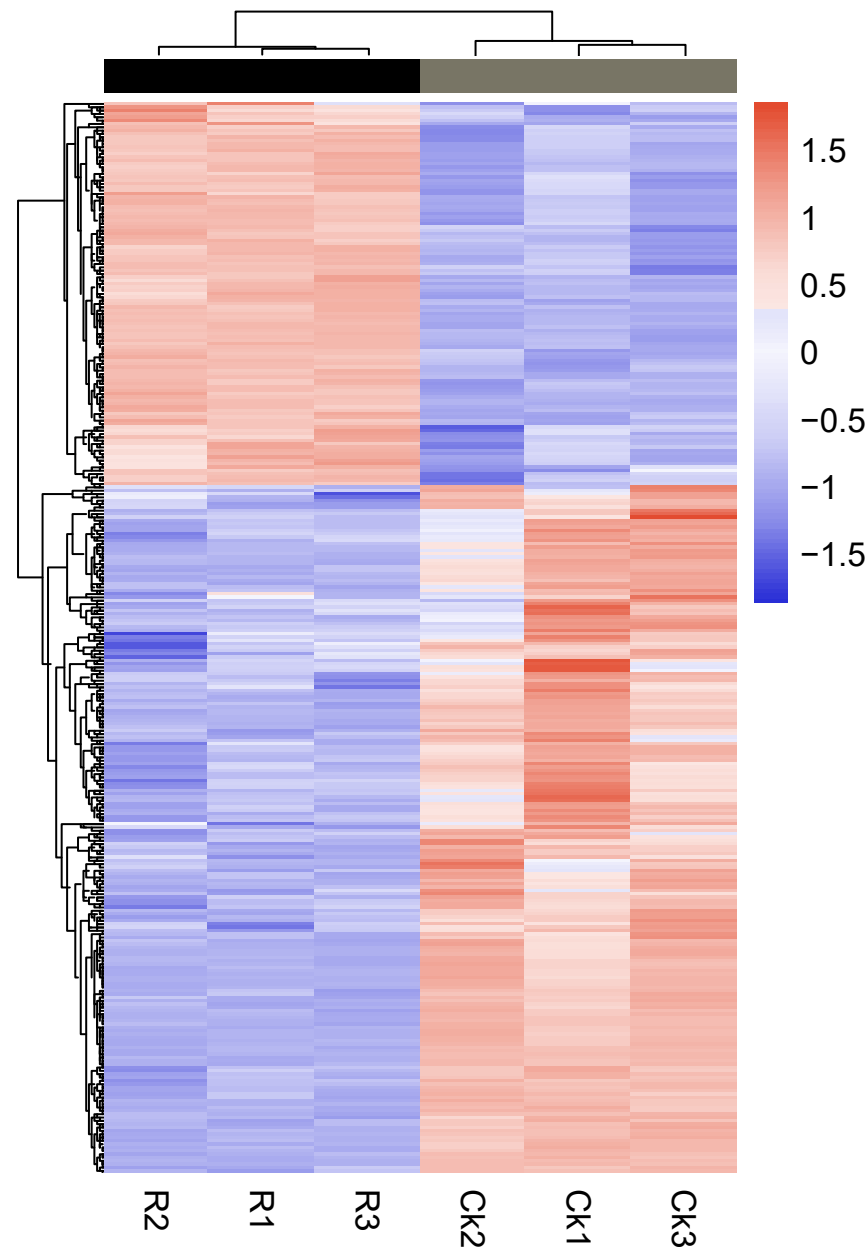

**(c)** Volcano Plot

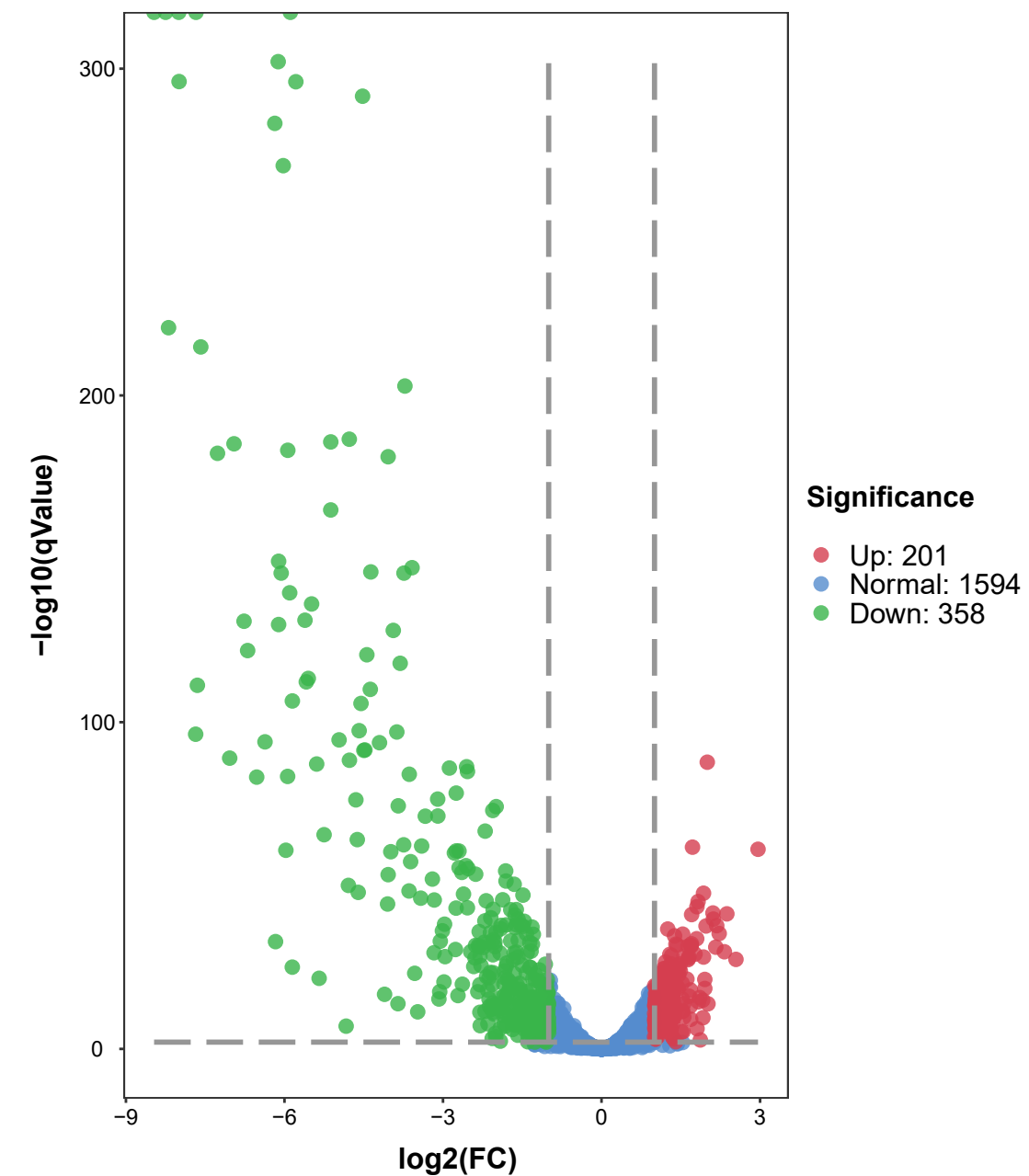

Supplement: Supplementary file 1 [file ijms-22-10036-s001.zip › ijms-1353640-supplementary/ijms-1353640-SM final/ijms-1353640-SM 2/Supplementary Figure/Supplementary.Fig1abc.pdf]

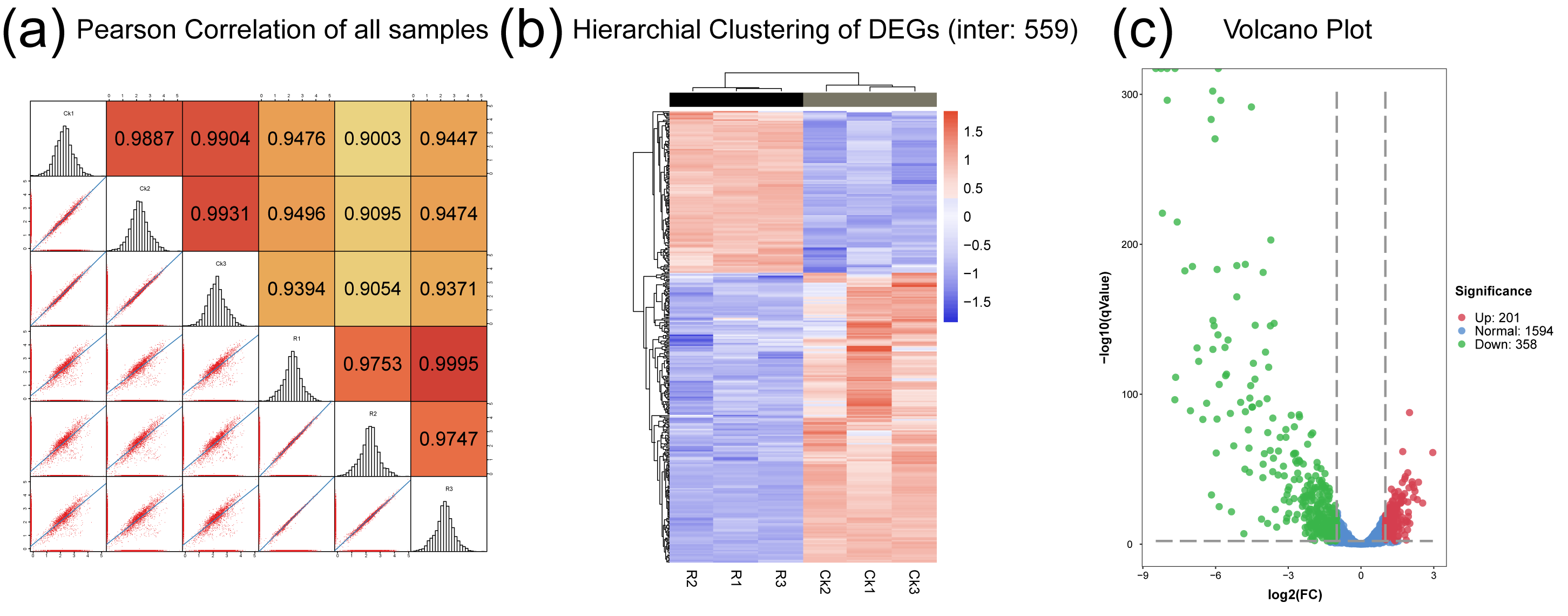

Supplement: Supplementary file 1 [file ijms-22-10036-s001.zip › ijms-1353640-supplementary/ijms-1353640-SM final/ijms-1353640-SM 2/Supplementary Figure/Supplementary.Fig1abc.tif]

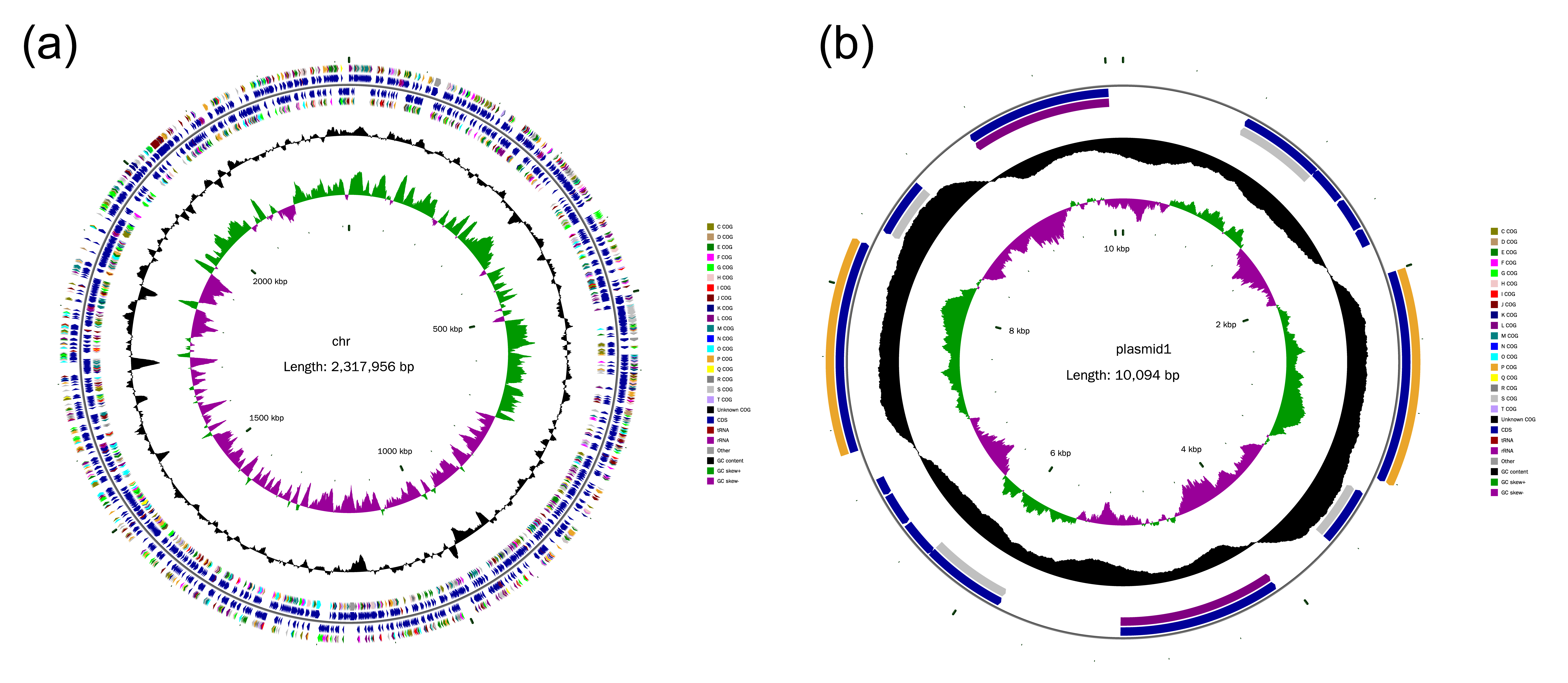

Supplement: Supplementary file 1 [file ijms-22-10036-s001.zip › ijms-1353640-supplementary/ijms-1353640-SM final/ijms-1353640-SM/Figure.1_genomic_chr&plasmid-01.tif]

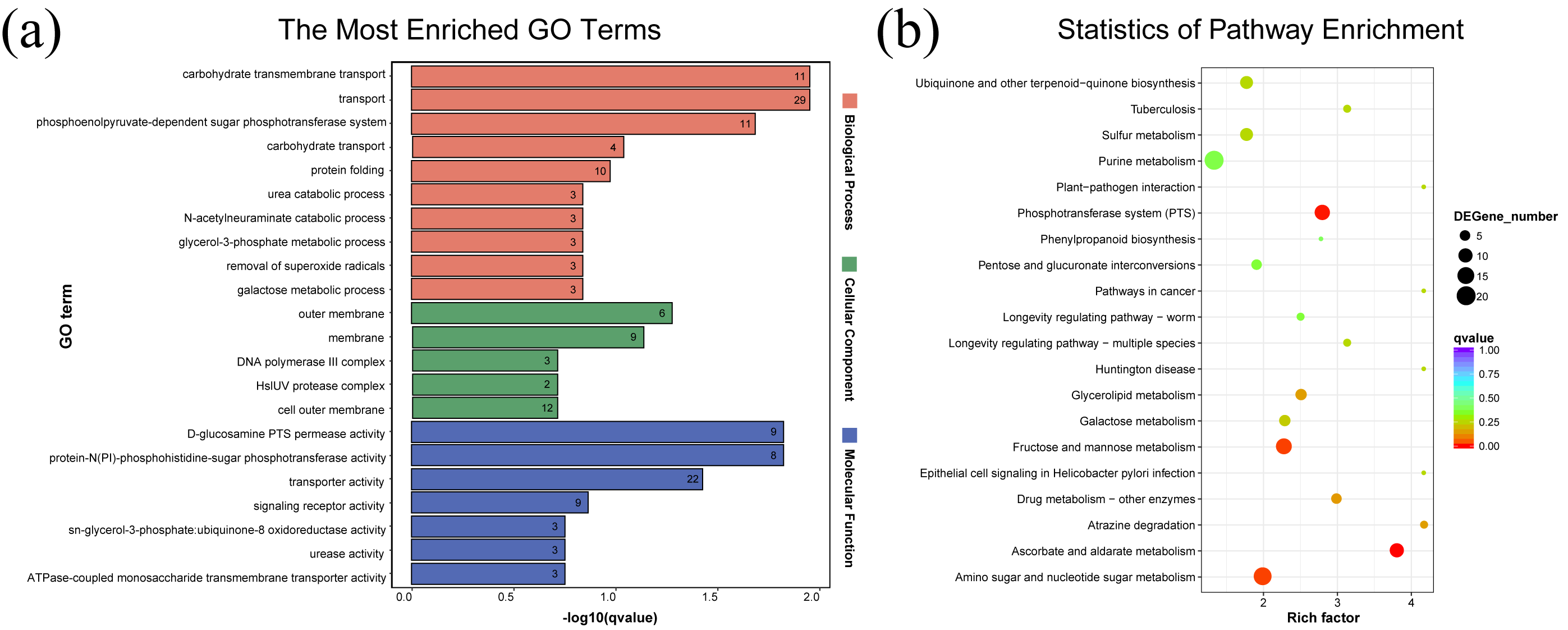

Supplement: Supplementary file 1 [file ijms-22-10036-s001.zip › ijms-1353640-supplementary/ijms-1353640-SM final/ijms-1353640-SM/Figure.2_transcriptome_summery.tif]

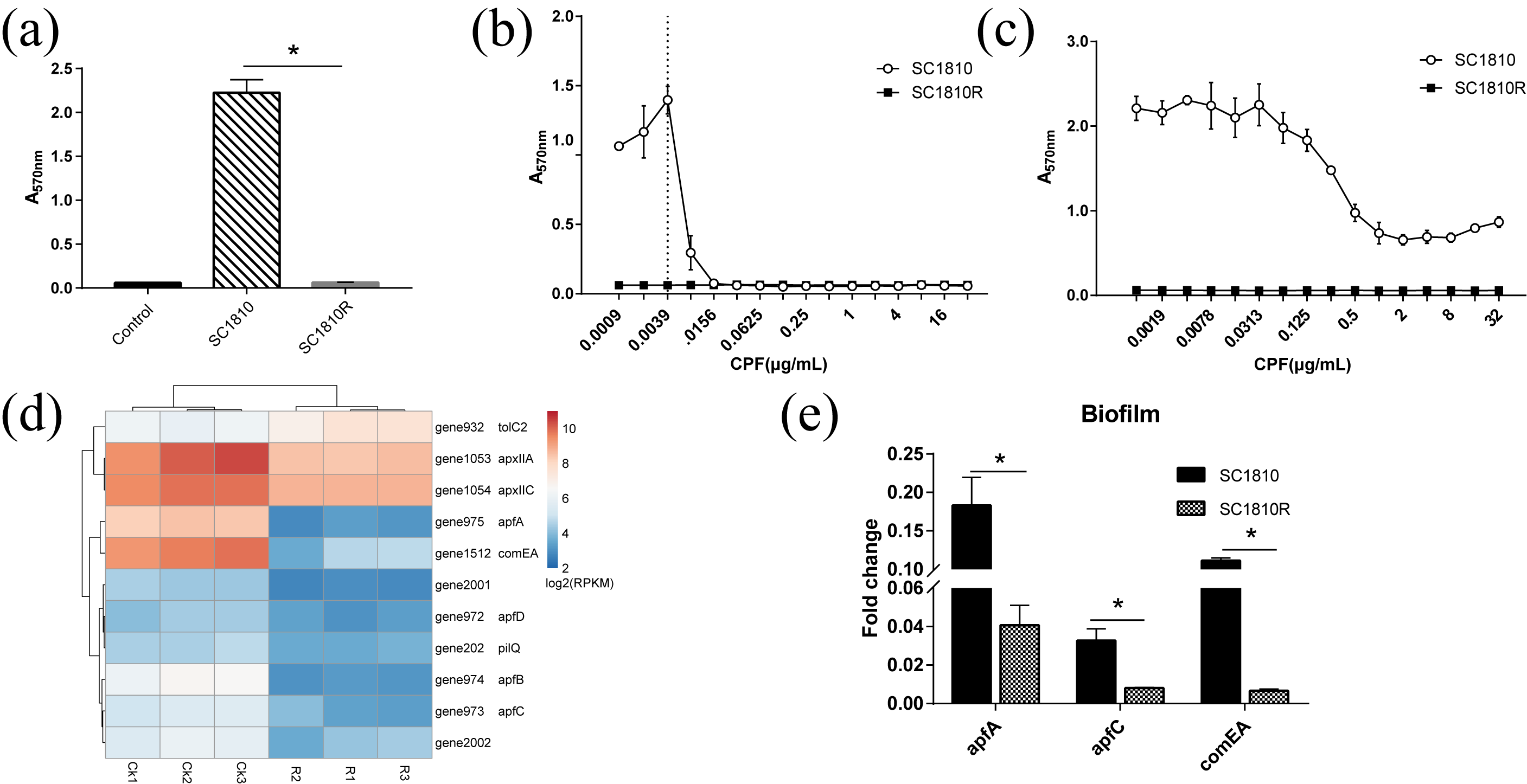

Supplement: Supplementary file 1 [file ijms-22-10036-s001.zip › ijms-1353640-supplementary/ijms-1353640-SM final/ijms-1353640-SM/Figure.3_Biofilm_summry.tif]

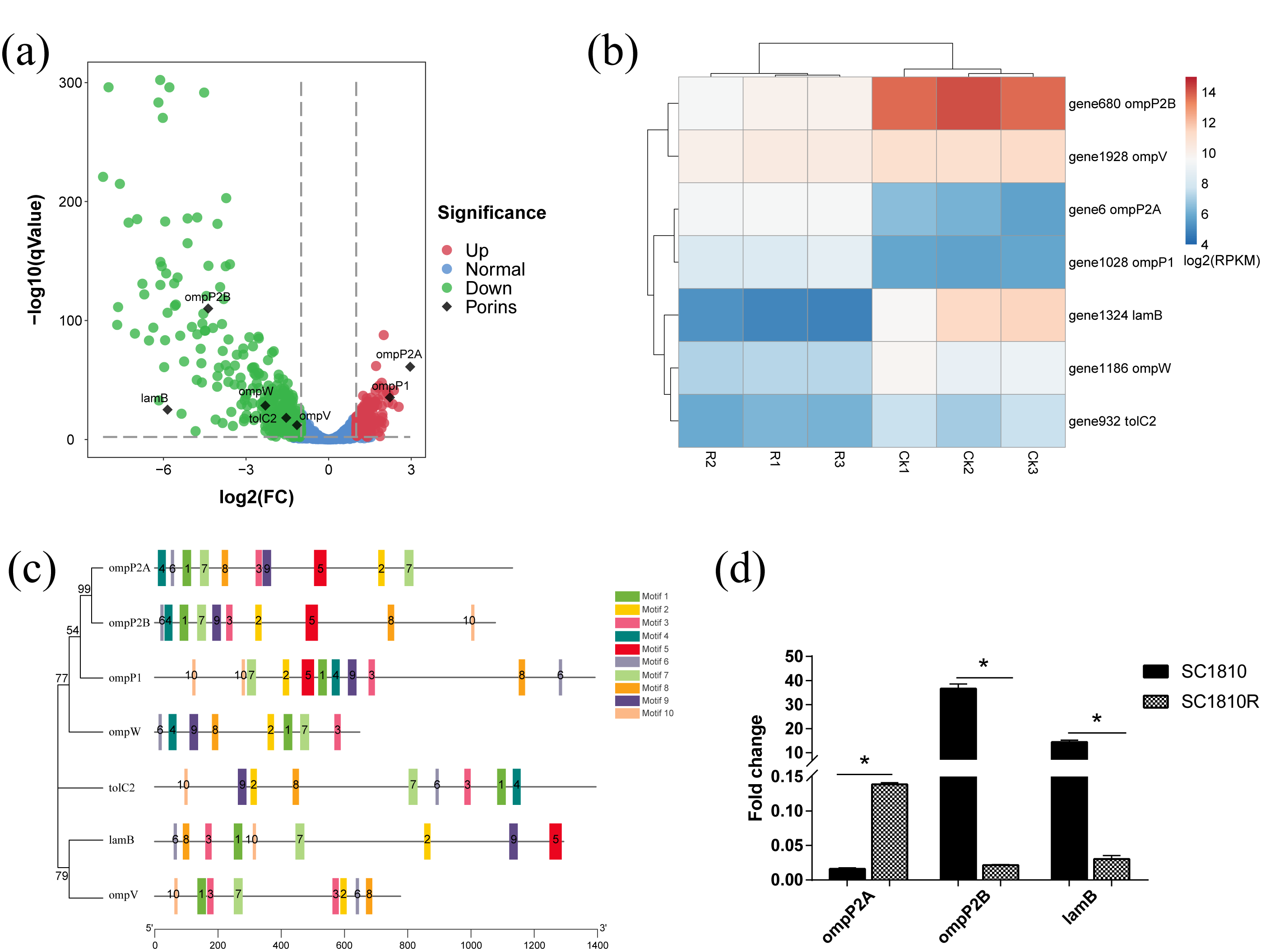

Supplement: Supplementary file 1 [file ijms-22-10036-s001.zip › ijms-1353640-supplementary/ijms-1353640-SM final/ijms-1353640-SM/Figure.4_porin_summry_new.tif]

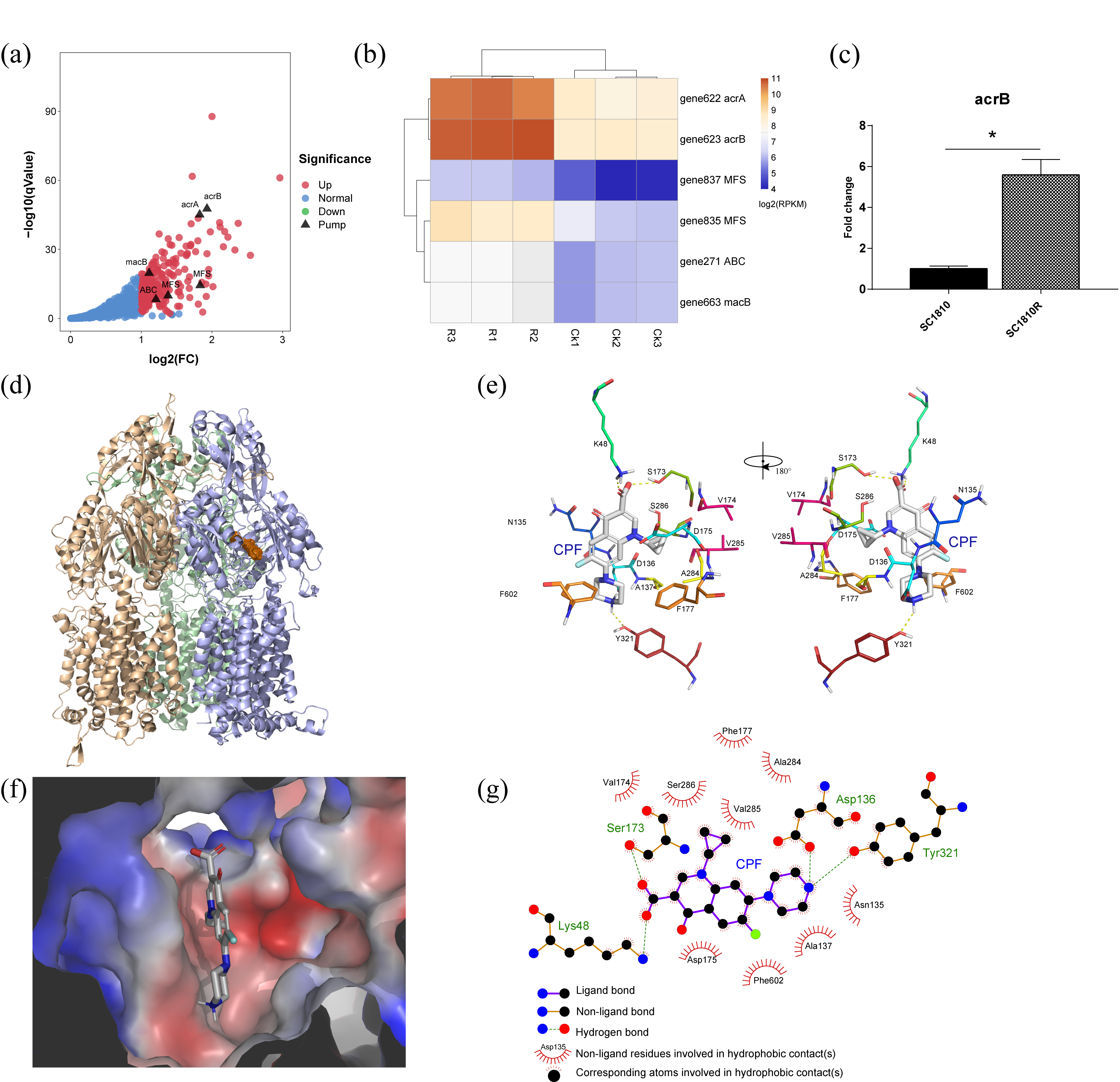

Supplement: Supplementary file 1 [file ijms-22-10036-s001.zip › ijms-1353640-supplementary/ijms-1353640-SM final/ijms-1353640-SM/Figure.5_pump_summry_ALL.tif]

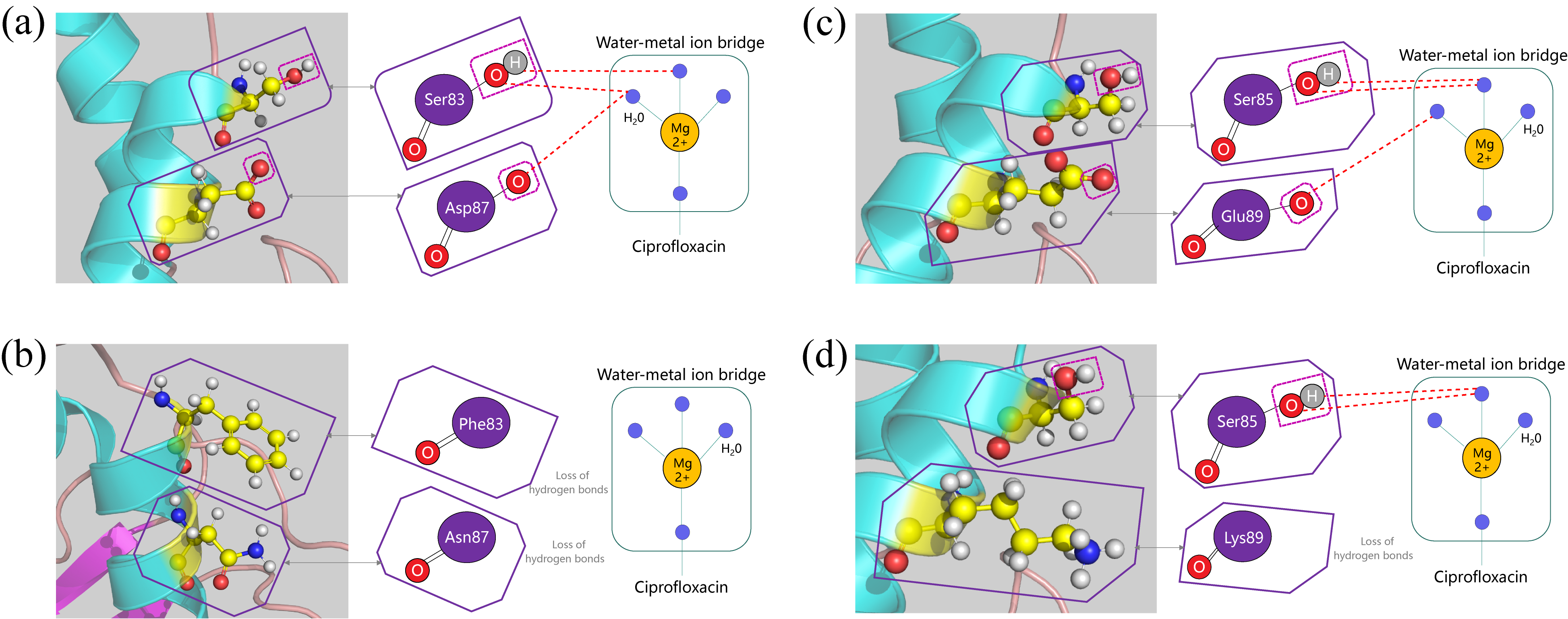

Supplement: Supplementary file 1 [file ijms-22-10036-s001.zip › ijms-1353640-supplementary/ijms-1353640-SM final/ijms-1353640-SM/Figure.6_mutant(gyrA&parC)_dpi150.tif]

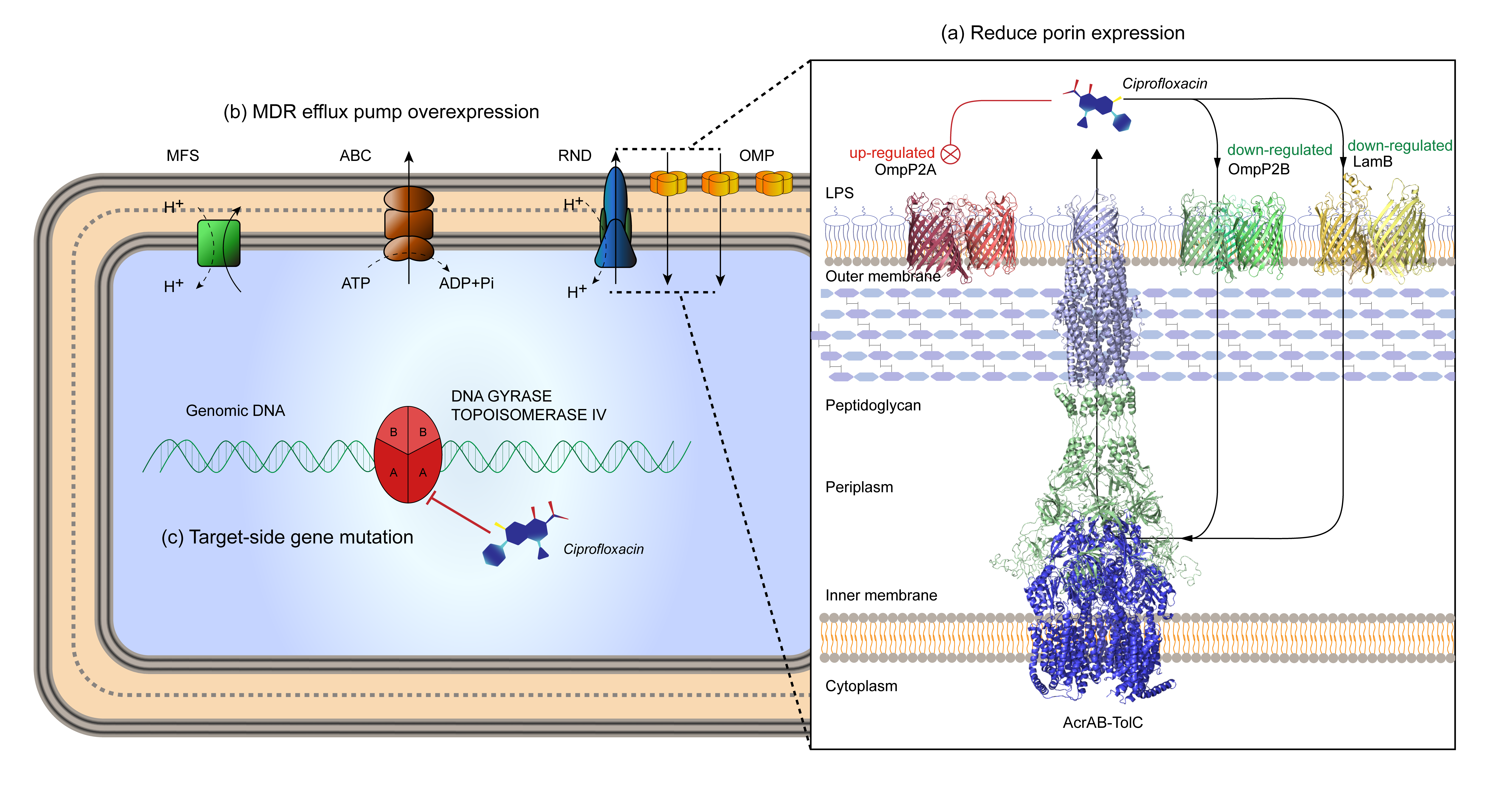

Supplement: Supplementary file 1 [file ijms-22-10036-s001.zip › ijms-1353640-supplementary/ijms-1353640-SM final/ijms-1353640-SM/Figure.7_conclusion.tif]
